# Testis-Sparing Approach in Paratesticular Leiomyoma: A Rare Case Navigating From Diagnosis to Targeted Treatment Planning

**DOI:** 10.7759/cureus.75234

**Published:** 2024-12-06

**Authors:** Annem Haritha, Sri Balram A, N. Rama Murthy

**Affiliations:** 1 College of Medicine, Mamata Academy of Medical Sciences, Hyderabad, IND; 2 Department of Urology, Mamata Academy of Medical Sciences, Hyderabad, IND; 3 Department of Urology, Mamata Medical College, Khammam, IND

**Keywords:** histopathology, leiomyoma, paratesticular tumors, radical orchiectomy, testis sparing approach

## Abstract

In this case study, we describe a 46-year-old male presenting with a palpable, gradually enlarging scrotal mass persisting over three to four years, ultimately diagnosed as paratesticular leiomyoma, who underwent enucleation of the tumor with no signs of recurrence, two years after surgery. This report underscores the significance of accurate diagnosis to avoid unnecessary treatment. We also emphasize the sequential events and findings, supported by relevant literature review, that contributed to establishing the correct diagnosis and guiding appropriate treatment decisions.

## Introduction

Leiomyomas are benign, slow-growing tumors of smooth muscle origin, capable of arising in various locations throughout the body [1]. Involvement of the male genitourinary tract is uncommon, with scrotal leiomyomas being an exceedingly rare occurrence. It generally presents as an asymptomatic, painless, palpable scrotal mass [1]. They are most commonly localized within the testis, epididymis, spermatic cord, subcutaneous tissue, tunica albuginea, and scrotal skin. Only a few cases have been reported where they originated in the paratesticular region without the involvement of paratesticular structures [1]. It needs surgical exploration for histopathological confirmation [2]. Paratesticular tumors have histopathological variants, including lipomas, adenomas, leiomyomas, fibromas, hemangiomas, neurofibromas, and cystadenomas, with leiomyomas being extremely rare [3]. Leiomyomas generally present as well-defined masses with a whitish-grey capsule, making surgical excision a primary treatment option [3]. In cases where there is concern for testicular malignancy or if the tumor is inseparable from the testis, a radical orchiectomy may be considered [3].

## Case presentation

**Figure 1:**
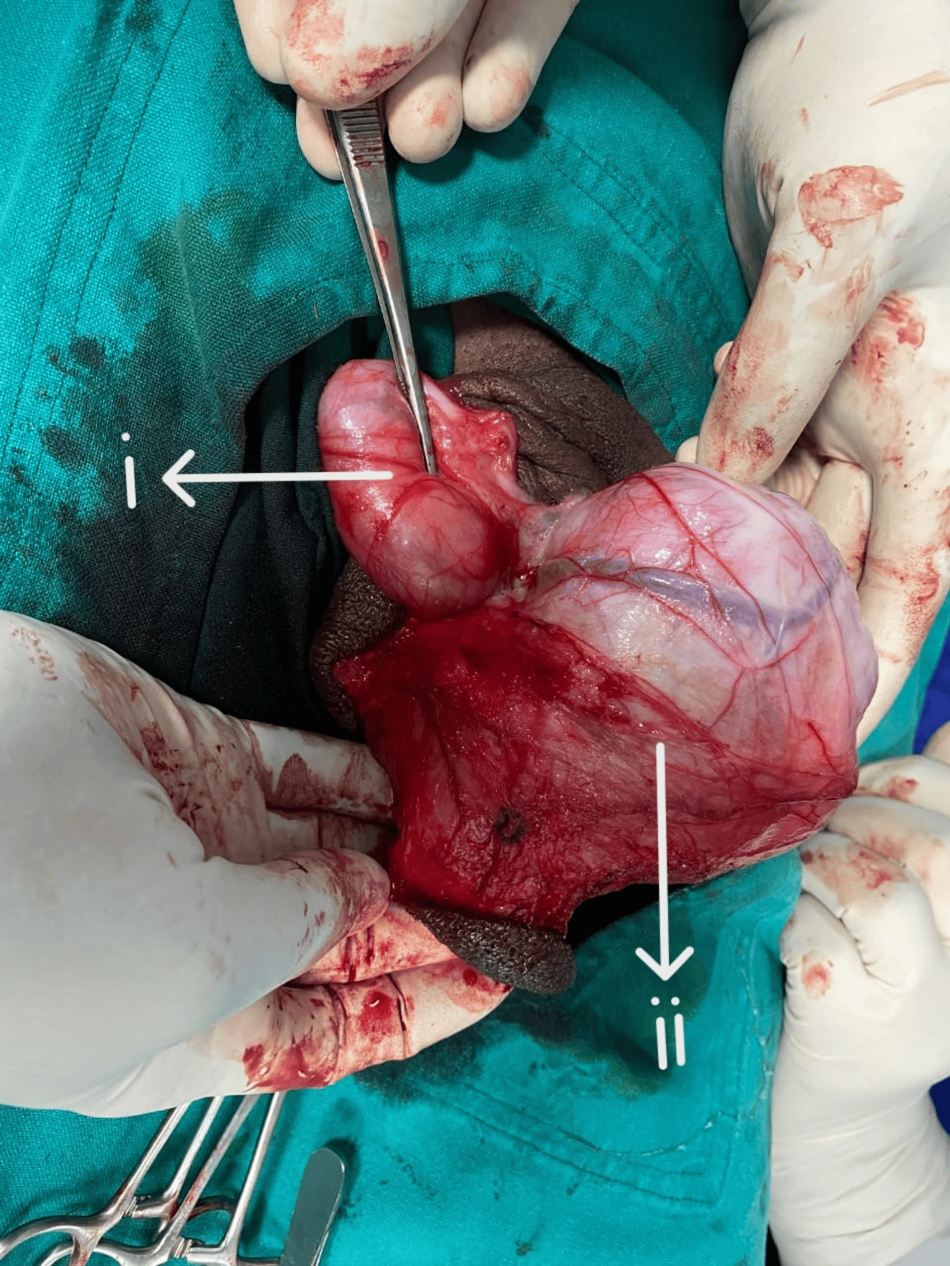
Intraoperative image showing (i) testes separate from the (ii) well-encapsulated tumor

**Figure 2:**
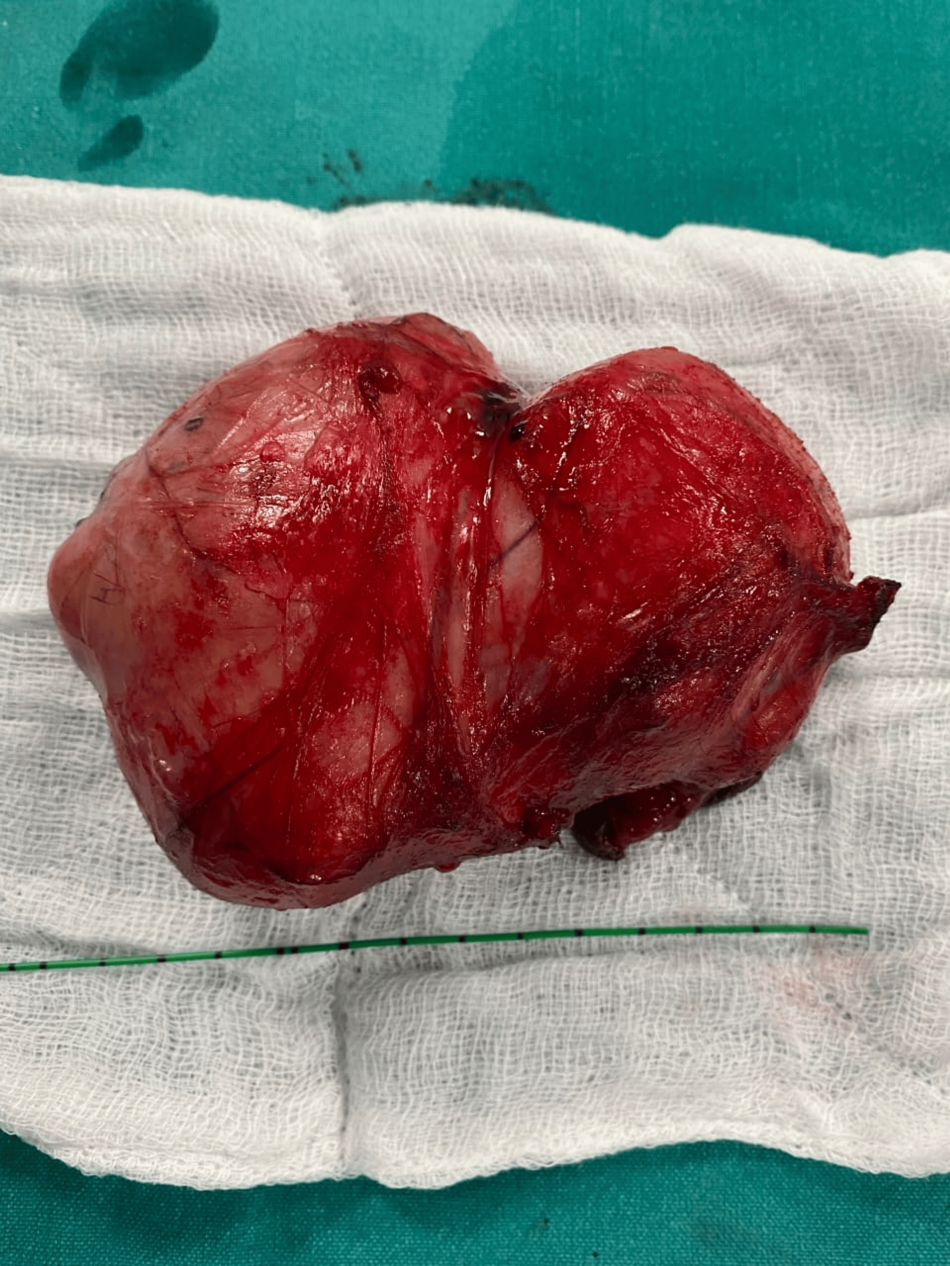
Post-operative specimen tumor enucleated in toto

**Figure 3:**
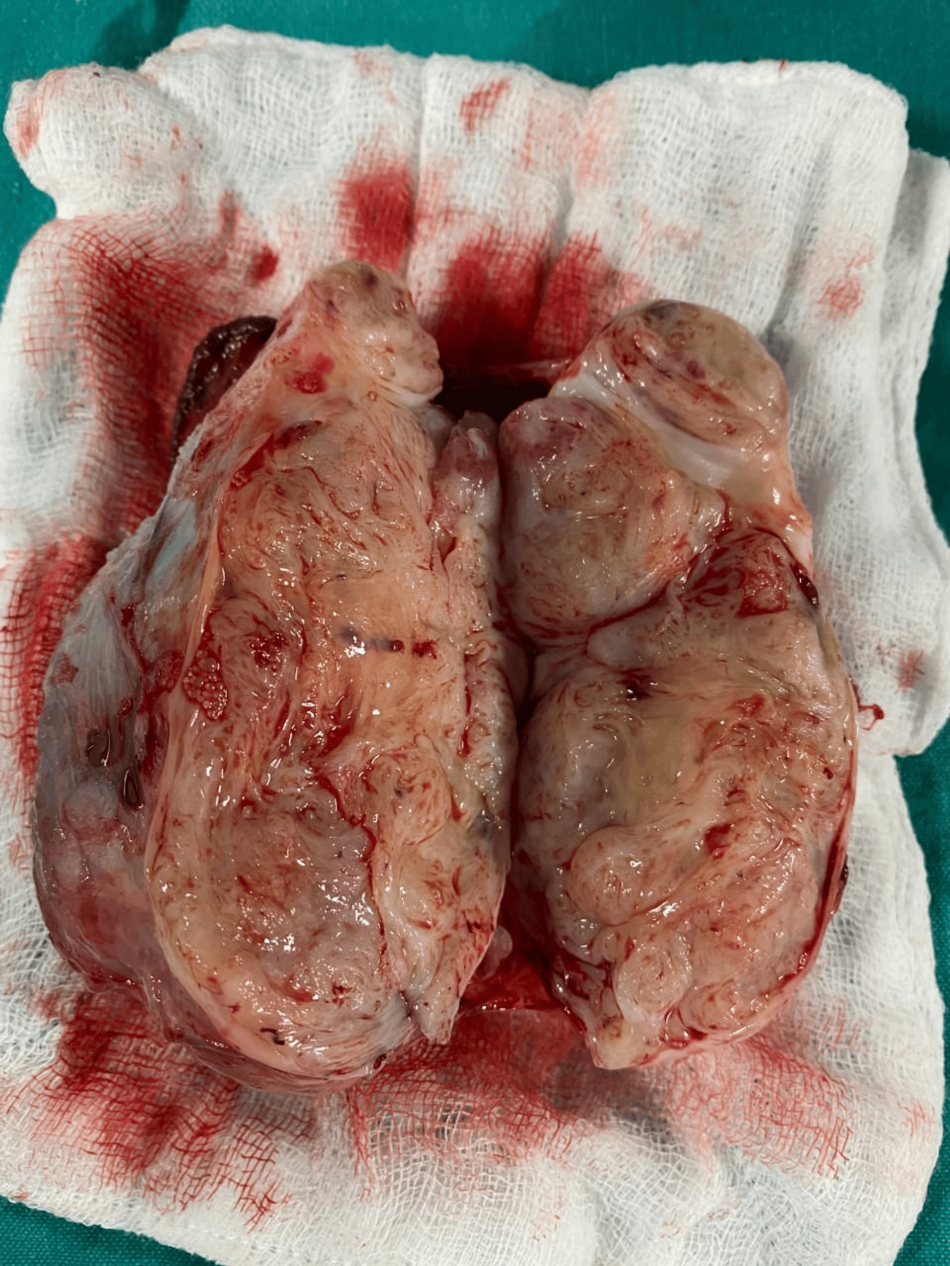
Post-operative specimen cut section, showing a uniform, firm mass with no areas of necrosis

A 46-year-old married man with two kids presented with a right-sided scrotal mass with heaviness in the scrotum, the patient himself perceiving it as an extra testicle, gradually growing in size for the past four years. He has no additional complaints. He has no comorbidities. Upon examination, a round, regular mass was felt separable from the right testis, non-tender and non-reducible, while the left side was normal, with no palpable lymph nodes on either side. Based on the above history and examination findings, testicular tumor, paratesticular tumor, though unlikely, and polyorchidism were considered as differentials. Further evaluation and scrotal ultrasound revealed a heterogeneously hypoechoic lesion of 10x4 cm in the right scrotum separate from the testis suggestive of a paratesticular tumor, ruling out the possibility of extremely rare polyorchidism. Tumor markers, including alpha-fetoprotein, human chorionic gonadotropin (HCG), and lactate dehydrogenase (LDH), were within normal limits. CT thorax, abdomen, and pelvis showed no features suggestive of metastasis or lymph node involvement. Considering the age of the patient and the size of the tumor, he was explained the possibility of orchiectomy or testis-sparing surgery based on intraoperative findings, and the patient’s consent for high inguinal orchiectomy was taken. The inguinal approach was preferred, and intraoperatively, the mass was identified to be well-circumscribed and well-separated from the testis and cord structures (Figure 1). The tumor was covered by a glistening grey-white capsule, distinct from the testis, with no signs of necrosis or hemorrhages and no lymph node involvement, supporting a very high likelihood of a benign lesion. Enucleation of the mass was done by resecting the tumor in toto (Figure 2). A sample was sent for histopathological examination (Figure 3), which revealed a benign spindle cell lesion suggestive of leiomyoma, and the diagnosis of paratesticular leiomyoma was established. The patient was under regular follow-up, and two years post-surgery, the patient had no signs of recurrence.

## Discussion

Most paratesticular lesions are benign cystic formations of the epididymis, such as cysts and spermatoceles, or scrotal fluid collections, including hydroceles and pyoceles. They may also present as inflammatory conditions, like acute and chronic epididymitis, or as hernias. Primary solid neoplasms within paratesticular tissues are, however, uncommon [4]. As the tumor is usually asymptomatic and grows bigger slowly [5], many patients postpone seeking treatment until the tumor grows significantly, leading to poor cosmetic outcomes [3]. Our patient noted a gradual enlargement of a scrotal mass over a span of approximately four years prior to seeking medical attention. Although a testicular tumor was considered unlikely based on ultrasonography findings, given the mass's distinct paratesticular location separate from the testes, the possibility of a paratesticular malignancy remained on the differential diagnosis, though less probable due to the benign characteristics noted during assessment and the absence of associated lymphadenopathy. Given the strong clinical suspicion of a benign paratesticular lesion, a pre-surgical biopsy or fine-needle aspiration cytology (FNAC) was omitted to avoid unnecessary invasiveness. However, it's acknowledged that establishing a precise preoperative diagnosis is advantageous to prevent overtreatment resulting from misdiagnosis. Therefore, we deemed the intraoperative findings crucial for guiding further management and preparing for a high inguinal orchiectomy with appropriate patient consent. An inguinal approach was preferred for testicular exploration, though a trans-scrotal excision of the mass could have been considered under absolute certainty of its benign nature [2]. The intraoperative findings, characterized by the absence of adhesions or invasive features and minimal vascularity, suggested a lower probability of malignancy. However, to exclude malignancy, an intraoperative frozen section after excision of the mass can be done for confirmation [1], although it was not done in our case due to resource limitations. In cases where benign lesions are confirmed, simple enucleation is preferred over radical orchiectomy to minimize complications and avoid unnecessary tissue removal. Although immunohistochemistry was not performed in this instance, its potential role in confirming leiomyoma diagnosis is acknowledged in comprehensive diagnostic protocols.

## Conclusions

Therefore, this case emphasizes the critical role of each phase, from history and examination findings, imaging studies, and intraoperative observations, to tailor treatment strategies and determine the appropriate level of intervention for the tumor. It underscores the significance of histopathology in confirming the diagnosis. Our management approach reflects a thorough consideration of clinical findings, relevant factors, and insights from the literature review, especially given the rarity of this presentation. Accurate diagnosis is paramount to avoid unnecessary treatment escalation in such cases.
